# Energy Absorption Capability of Thin-Walled Prismatic Aluminum Tubes with Spherical Indentations

**DOI:** 10.3390/ma13194304

**Published:** 2020-09-26

**Authors:** Miroslaw Ferdynus, Patryk Rozylo, Michal Rogala

**Affiliations:** Department of Machine Design and Mechatronics, Faculty of Mechanical Engineering, Lublin University of Technology, Nadbystrzycka Str. 36, 20-618 Lublin, Poland; michal.rogala@pollub.edu.pl

**Keywords:** crashworthiness indicators, energy absorbers, thin-walled structures

## Abstract

The paper presents the results of numerical tests of impact and energy absorption capacity of thin-walled columns, subjected to axial impact loading, made of aluminum alloy, and having a square cross-section and spherical indentations on their lateral surfaces. The numerical models were validated using an experiment that was conducted on the Instron CEAST 9350 High Energy System drop hammer. Material properties of the applied aluminum alloy were determined on the basis of a static tension test. The crushing behavior of the columns and some crashworthiness indicators were investigated. On the basis of the results of the conducted analyses, conclusions were drawn about the most beneficial design/constructional variants in terms of achieved crashworthiness parameters.

## 1. Introduction

Since the beginning of the 1960s, safety regulations in the automotive industry have stimulated the development of new vehicle concepts with a high level of passive safety. It was connected with the necessity of conducting research on the crumple zone of vehicles. A very important element of that zone is the so-called crash box, which is intended for absorbing impact energy and protecting the longitudinal members of the vehicle up to a speed of 15–20 km/h.

At a comparable time—in the 1960s—the problem of impact load of metal tubes, especially of circular and square cross-section tubes, appeared in the article [1,2]. However, due to the underdevelopment of numerical methods, subsequent work started to appear only about twenty years later. Thin-walled metal tubes, particularly those of square, rectangular, or circular cross-section, are widely used as energy absorbers since they are relatively inexpensive and efficient for absorbing energy. The behavior of this type of structure during axial, oblique, and lateral crushing has been extensively studied over the last decades. These studies are reflected in numerous publications, among which the review articles contain more and more titles [1,2,3], as well as in the emergence of valuable book items [4,5]. The deeper considerations taking into account the basis of analytical description and experimental research on thin-walled constructions were presented by Abramowicz [6].

There are many types of energy absorbers that are quoted in the literature [1], their design often depends on the type of load they will have to deal with: whether it is axial, oblique, or lateral. Various cross-sectional shapes are considered (circle, square, rectangles, or polygons) [7], but also intensive research is being carried out on multicell, multicornered, and bitubal structures. Studies are conducted on hollow structures, but also on filled structures, for example, with foam or honeycomb filling [8,9]. In spite of such advanced research on more advanced structures, simple tubular structures are still widely used as energy absorption systems in the automotive industry due to their high energy absorption capability, easiness of fabrication, and cost-effectiveness.

For thin-walled members subjected to axial compression, working as energy absorbers, it is necessary to develop a concept that would promote a progressive buckling mechanism, stimulating at the same time the highest energy absorption capacity with acceptable energy absorption indicators. One of the possible engineering solutions is to apply a trigger (usually in the form of a notch or dent) to release the desired crushing mechanism. The term “trigger” indicates some kind of mechanical deformation which is favored because of the presence of intentionally placed sites of stress concentration and plasticization, in a material science sense. If we opt for a simple form of thin-walled structures, the type of the trigger used and its geometric parameters have a key significance for their energy-absorbing performance.

There are many publications on the problem of energy absorption of thin-walled tubes, but only in a few of them, the trigger mechanism has been properly appreciated [10,11], and after all, the role of the trigger, although limited to initiating the crushing process, has an undeniable impact on the achieved parameters, especially Crush Load Efficiency (CLE). In this work, we would like to focus on the influence of a new type of trigger, its geometric parameters, and the energy absorption indicators achieved to fill the research gap we see.

Although there has been considerable research to date in the development of various energy-absorbing structures (EAS), the main disadvantages of most structures are that they show high initial peak crushing force (PCF) and low stroke efficiency (SE). The greatest challenge in constructing EAS is to design them in such a way as to reduce the intensity of impact-induced deceleration, while maximizing the energy absorbed. An attempt to solve this problem can be found, among others, in Reddy’s work [12], where the authors focused on this problem. The numerical research and proposals of interesting ideas were approved on the basis of experimental data.

A very valuable publication is the work of Karagiozova and Jones [13], where the dynamic elastic–plastic buckling of thin-walled square tubes was studied from the point of view of the propagation of the elastic–plastic stress wave that originated from axial impact load.

A quite promising approach to increase energy efficiency and impact resistance of thin-walled structures was proposed by Zhang et al. in article [14], by introducing a thickness gradient in the cross-section. The experimental tests were first performed for square tubes with two types of thickness distribution, and only then finite element numerical (FE) analyses were performed to confirm the results of the experiment. Relatively recently, Yang et al. [15] presented a novel type of tubular structure in which predesigned ellipsoidal dimples were introduced into conventional circular tubes made of brass powder using the 3D printing technique. Dimples were periodically located, both along the circumference and height. 

Interesting studies on the effect of triggering on the energy absorption capacity of axially compressed aluminum tubes are presented by Lee et al. [10], who examined aluminum tubes with prismatic dents, placed in different configurations on the side surfaces of the tested columns. On the other hand, the influence of triggers in the form of cylindrical dents located at the edges of columns was analyzed in the articles [16,17], where advanced numerical studies with experimental validation have been demonstrated. An experimental investigation showing the effect of induced imperfections on the symmetric progressive buckling of thin-walled square mild steel tubes was presented by Marshall et al. [18]. The imperfections included a circular hole, indentations of various shapes, and combinations of a hole positioned centrally in an indentation. Unfortunately, in that work, which is already old, only the influence of imperfections on the value of PCF called by the authors as ultimate buckling load is shown. Energy-absorbing indicators were not calculated.

A few years later, Cheng et al. [19] also performed an experimental research study. The objects of the research were aluminum alloy extrusions with centrally located through-hole discontinuities. Three different types of geometrical discontinuities, namely, circular, elongated, and elliptical holes, were fabricated. The columns had a square cross-section. In addition to the peak crushing force (PCF), crash force efficiency (CFE) and specific energy absorption (SEA) indicators were calculated for three groups of specimens (large, medium, and small discontinuities). The specimens were crushed using the quasi-static method. 

Estrada et al. [20], in their paper, analyzed the effect of discontinuity size on energy absorption performance of steel square profiles. Cut-outs made in the profiles were of square, rectangular, or diamond shape. According to results obtained in the ‘perfect’ profile, the implementation of quadrilateral cut-outs showed a significant reduction on initial peak loads.

As mentioned above, the literature on tubular structures is quite extensive: from analytical considerations through experimental work to FE simulations. However, relatively little attention has been paid to the triggering mechanism and the influence of different types of triggers (especially in the form of embossing) on the energy-absorbing indicators achieved. This is shown in the review of axial impact-loaded tubular structures published by S. Yuen and G. Nurick [3], who analyzed the energy-absorbing properties and behavior of such structures with different types of imperfections and with the possibility of fillers. Within the analyzed studies, no crash initiator in the form of spherical indentation in the side walls was found. For this reason, this topic has been taken up. The first results based only on numerical simulations, a relatively small number of models, were promising and were presented in the conference materials [21]. It was decided to continue the research and the number of model variants was extended and experimental verification was conducted, which resulted in this publication.

The proposed new type of trigger, in the form of spherical indentations (concave and/or convex) placed on the side surfaces, extends the range of known solutions. The present study focused on the analysis of the behavior of classical square tubes, with particular emphasis on the influence of the new type of trigger on the energy absorption indicators achieved (triggering effect). The main objective of the research was to conduct an extended parametric study on the influence of some geometrical parameters, mainly depth and diameter of the indentation, on the columns’ crushing behavior and their ability to absorb energy.

## 2. Model of the Column with Indentations

**Figure 1:**
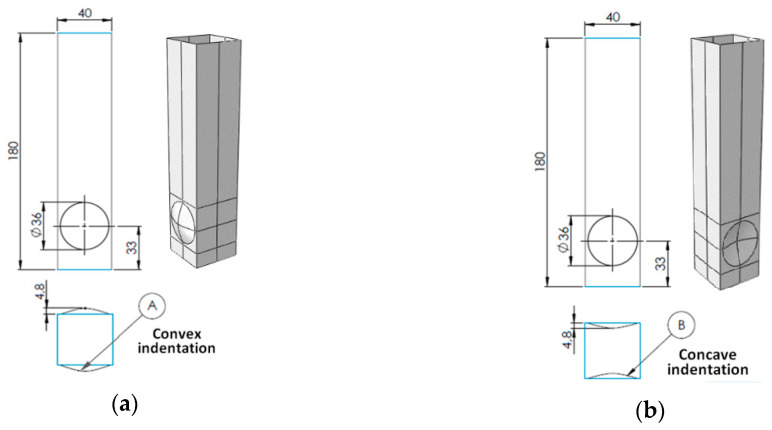

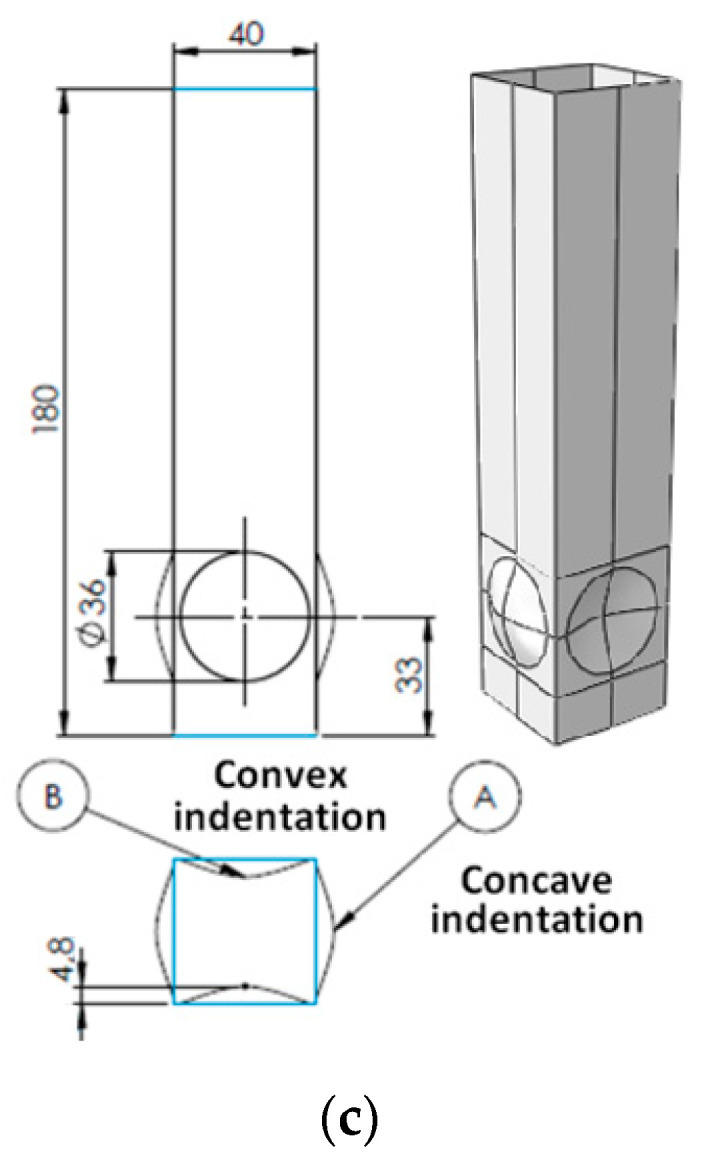
Column model with (**a**) convex spherical indentations—A, (**b**) concave spherical indentations—B, and (**c**) convex and concave spherical indentations—AB.

Column models differing in the trigger’s geometry (indentation diameter and depth) were designed in the Catia v5 package (Catia v5r20, Dassault Systemes Simulia Corporation, Velizy Villacoublay, France) (Generative Shape Design module) and imported into the FE system ABAQUS (in case of nonlinear dynamic or static analysis). Three groups of models were analyzed: A, with convex indentations; B, with concave indentations; and AB, with both concave and convex indentations. The models were designated by the symbols A/X/Y, B/X/Y, and AB/X/Y, where the first number X stands for the diameter of the indentation, while Y stands for the depth of the indentation expressed as a multiplication factor of the wall thickness (1.2 mm). In the study, the square section prismatic columns of dimensions 40 mm × 1.2 mm and height l = 180 mm were under investigation. Column models and the distance of indentations from the base (which was constant and connected to the placement of the nether half-wave in the buckling analysis) are shown in Figure 1.

## 3. Energy Absorbing Effectiveness—Crashworthiness Assessment Indicators

**Figure 2:**
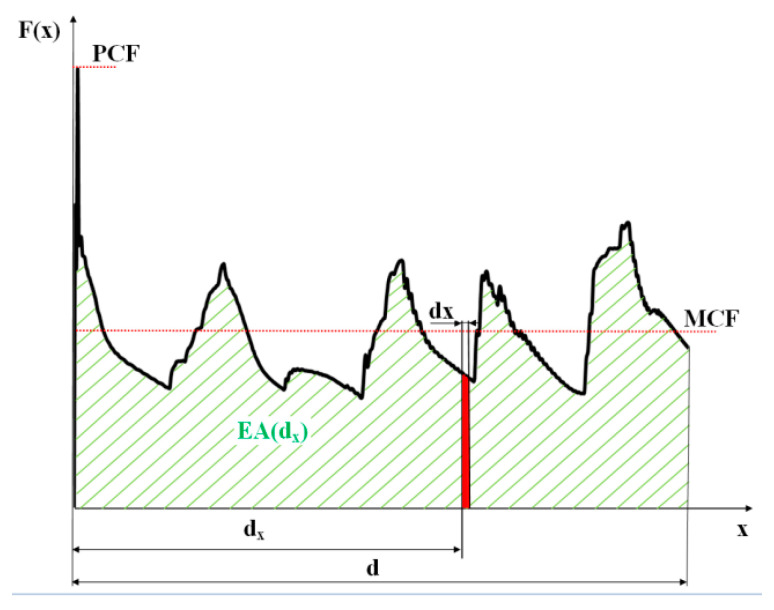
Exemplary load-shortening diagram of a thin-walled column under axial impact.

The crashworthiness efficiency of energy absorbers can be determined in many ways. There are several indicators of crashworthiness, and each evaluates the crush behavior of the Energy Absorbing Structure (EAS) taking into account different aspects [22,23]. The typical crushing load-shortening curve for a thin-walled, tubular member under axial impact is shown in Figure 2. The course of the graph in Figure 2 is characteristic of the so-called progressive buckling. The initiation of the crushing process usually corresponds to the peak crushing force (PCF), which can be reduced by a properly designed trigger. The load drop is related to the appearance of the plasticized zone and then the structure starts to fold. When the energy absorption capacity of this part of the member is exhausted, the load begins to increase until the appearance of plasticization in the next place. The process repeats itself until all the energy is absorbed or until the member is unable to absorb it, which is manifested by the appearance of large overloads.

This study presents only the most important equations, strictly describing crashworthiness indicators, while detailed information was presented in the previous study [24]. An indicator directly showing the absorption efficiency is the value of absorbed energy (EA—energy absorbed) given as relation (1), or specific energy (SEA; energy per mass unit) given as (2), where *m* is the mass of the absorber.

(1) $$\mathrm{EA}\left(d_{x}\right)=\overset{d_{x}}{\underset{0}{\int }}F\left(x\right)\mathrm{dx}$$

where d_x_ is the crushing distance and F(x) is the crushing force as a function of crush distance *x* (see Figure 2).

(2) $$\mathrm{SEA}=\frac{\mathrm{EA}}{m}$$

where m is the mass of the object.

The two aforementioned quantities (EA and SEA) show the amount of energy absorbed by EAS, but they do not reveal whether the process was efficient and whether the member’s capabilities were exhausted and to what extent.

There are several other factors that help to assess the energy absorption capacity and provide a deeper understanding of EAS’ crashworthiness properties. Since the reduction of the initial peak crushing force (PCF) is primarily desirable for biomechanical reasons, this factor is an extremely important indicator of the protective capabilities of the absorber. On the other hand, the ideal EAS should show a fairly regular distribution of the crushing force in relation to the crushing distance. Two further crashworthiness indicators allow to assess how close the structure’s crushing behavior is to ideal from the point of view of the protected person. These are the mean crushing force (MCF) and the crash load efficiency (CLE).

The mean crushing force (MCF; Figure 2) for a given crushing distance d_x_ is calculated as:

(3) $$\mathrm{MCF}=\frac{\mathrm{EA}\left(d_{x}\right)}{d_{x}}$$

The MCF is related to the shortening of the specimen during crushing. All analyses were carried out until all energy was absorbed (velocity = 0). MCF value was determined as the EA/U (U—maximum shortening) ratio. Crush load efficiency (CLE) is defined as the mean crushing force (MCF) to peak crushing force (PCF; Figure 2) ratio:

(4) $$\mathrm{CLE}=\frac{\mathrm{MCF}}{\mathrm{PCF}}\cdot 100\%$$

The CLE should be as high as possible. The ideal energy absorber reaches a theoretical value of 100%, unfortunately in practice this is not realistic.

Another crashworthiness indicator is stroke efficiency (SE), which represents the deformation capacity of an absorber and it is defined as follows:

(5) $$\mathrm{SE}=\frac{U}{L_{0}}$$

where L_0_ is the initial length of the member (mm) and U is the maximum shortening (crushing distance) of the member.

The most desirable value of the SE factor is the highest, corresponding to the highest value of the crushing distance. This is one of the basic indicators of crushing performance. The ideal structure during the crushing process should allow the entire available length of the member to be used to absorb the impact energy, if this energy is large enough. A combination of the CLE and the stroke efficiency (SE) was proposed by Hanssen et al. [8] as total efficiency (TE) to assess the whole performance of an energy absorber. It can be expressed as:

(6) TE=CLE×SE

## 4. FE Model 

**Figure 3:**
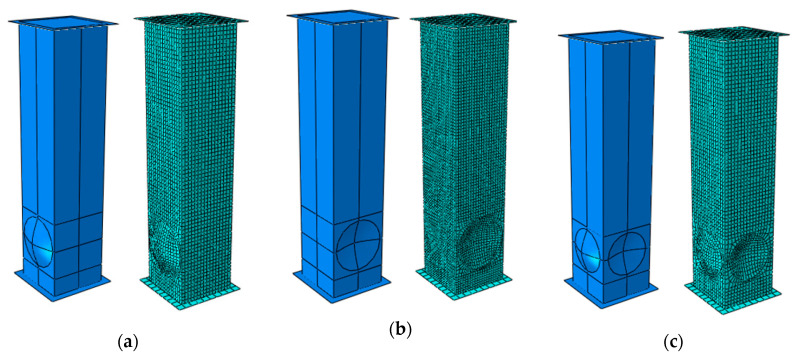
Finite element models with (**a**) convex spherical indentations—A/36/4, (**b**) concave spherical indentations—B/36/4, and (**c**) convex and concave spherical indentations—AB/36/4.

The analysis of the crushing behavior of the tested columns was performed on the basis of FE simulations. The explicit FE analysis was performed using the ABAQUS software. The numerical model contained rigid plates between which the tested column was placed (Figure 3). The column model was created using 4-node shell elements with reduced integration. The rigid plates were modeled using 4-node bilinear, quadrilateral, rigid elements. The global size of elements both in the rigid plates and in the columns was 2.5 mm. The column was impacted with a kinetic energy (E) of 1715 kJ, corresponding to a mass (m) of 70 kg dropping at an initial speed (V_0_) of 7 m/s.

The columns were assumed to be made from the aluminum alloy EN AW6063-T6, the properties of which were determined in the static tension test of several specimens obtained from a column, from which the models for stationary tests were made. The material properties of the aluminum alloy are presented in Table 1. As aluminum alloys do not show significant sensitivity to the strain rate [25], an advanced material model (elastic–plastic) was used, ignoring the effect of the strain rate, but taking into account the hardening during deformation. Due to the fact that the bottom and top plates were modeled as perfectly rigid, they were not given material properties.

In the preliminary analyses, both the case of the connection between the plates as a contact relation and *Tie* relation was considered. In both cases, the results obtained showed a similar behavior of the structure. Despite a large simplification with the use of *Tie* relation, the obtained results were adequate for experimental studies. In order to connect the rigid plates, with the end of cross-section of column (shell), *Tie* links were applied. In the rigid plates, reference points were created—upper and lower. The impact force was recorded in the lower point and the acceleration, speed, and displacement were recorded in the upper point. All degrees of freedom on the bottom rigid plate were locked, while on the top plate only vertical movement was possible. In order to create a homogeneously divided mesh, the partitioning technique was used. The contact domain was set up as the General Contact option (all with self). In the tangential plane, the contact properties of *Penalty* type were assumed, with a coefficient of friction equal to 0.2. The behavior in the normal plane was declared as the “*Hard Contact*” option. Constitutive relation does not cover the failure criterion. The Finite Element Method (FEM) numerical model is presented in Figure 3.

A numerical analysis of the crash of a column was conducted in two stages. In the first stage, an analysis of buckling was performed, which resulted in obtaining the buckling modes. In the second stage, the nonlinearly geometrical problem of impact was applied to a rigid plate of a given energy. In order to obtain credible results corresponding with the results of experimental tests, geometrical imperfections relating to the first buckling mode with an amplitude equal to 1/10 of the wall thickness were considered. A similar method of modelling such phenomena was presented in articles [26,27]. The analysis of ideal thin-walled structures in axial compression in ABAQUS (in case of nonlinear dynamic or static analysis) requires prior consideration of the geometric imperfection corresponding to the first form of buckling (which must be determined initially from linear buckling analysis). The buckling form in which the actual structure behaves, determined by the buckling analysis, is then implemented into the non-linear static or dynamic analysis by special procedures. For a column with identical dimensions and without indentations, FE comparative calculations were performed. This model was designated smooth model (SM).

**Figure 4:**
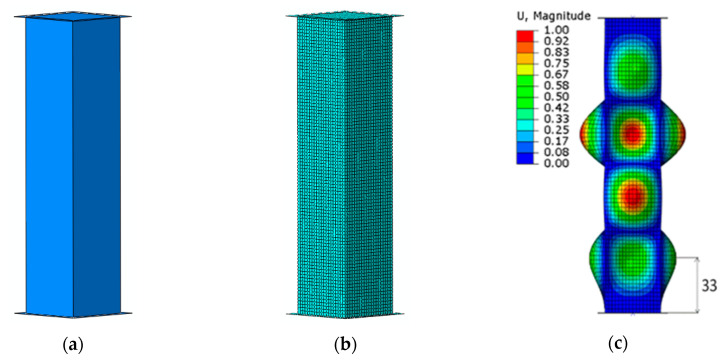
Object of study: (**a**) smooth model (SM), (**b**) FE model, and (**c**) first buckling mode.

Figure 4 shows the column shell model without indentations, the discrete model, and the first buckling mode of the smooth model. The position of the lower half-wave of the column determines the location of the indentation (trigger).

## 5. Experimental Verification of the Numerical Model 

**Figure 5:**
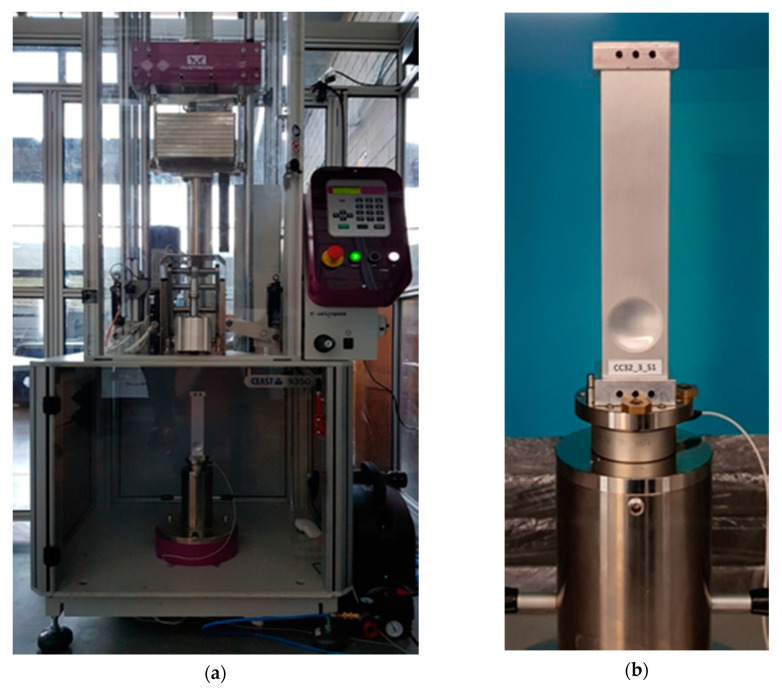
Test stand with specimen. (**a**) General view of the drop hammer rig (Instron CEAST 9350HES) and (**b**) a specimen fastened to the bottom plate of the rig.

Impact tests were carried out on the drop hammer rig, Instron CEAST 9350HES, the general view of which is shown in Figure 5a. For verification specimens, type B was selected, since its manufacturing does not pose technological problems (the material of the wall is pressed into the column, not outside of it). Specimens were made from standard aluminum tubes of square section 40 mm × 40 mm and wall thickness 1.2 mm. The analyzed tubes were produced only by extruding standard commercial profiles. Inside the profile, at a suitable distance from the edge (symmetry center of the die must be positioned 33 mm from the edge of the column), a special, divided die was located and then cold-stamping using a special rubber was performed. 

**Figure 6:**
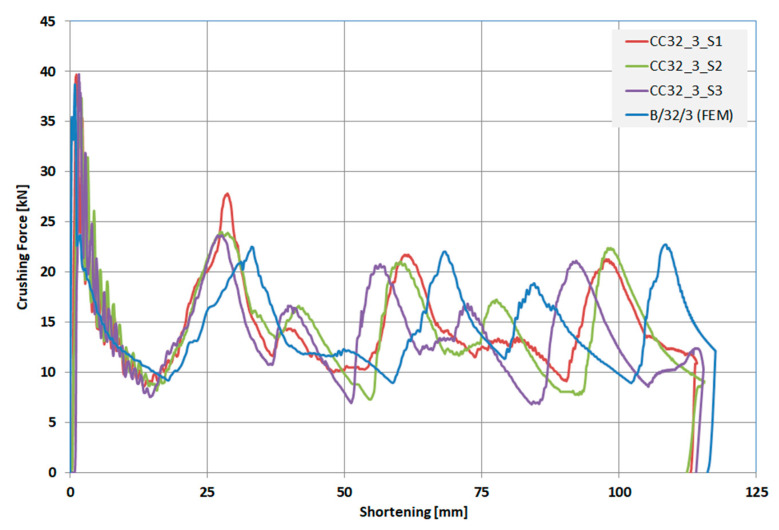
Load-shortening diagram. A comparison of experimental and numerical results for columns CC32_3_S1 to S3 and B/32/3 (Finite Element Method).

The specimens were denoted as CC32_3_S1 to S3, where CC meant concave, number 32 denoted the diameter of the indentation, and number 3 denoted the depth of the indentation. It was assumed that FEM models and experimental specimens will not be determined in the same way so that at first glance one can see whether it was a numerical model or a specimen for experimental studies, i.e., real objects. The use of a different separator in the designations additionally emphasized this. The need for other markings was particularly evident in the legend of Figure 6. The specimens were sealed with rectangular cubes with vents and mounted on a machine measuring table equipped with a piezoelectric force sensor (Figure 5b).

As a result of the conducted tests, load-shortening characteristics were obtained, which are shown in Figure 6 along with the course for the B/32/3 model, acquired as a result of FEM calculations. The dimensions of this model were the same as the aluminum specimens. 

The deformation of specimen S1 showed a similar peak force as specimens S2 and S3 (shown in Table 2, column 4). The PCF values were similar in Figure 6, but during the generation of the next fold, we observed a slight discrepancy in the crushing force value. 

**Figure 7:**
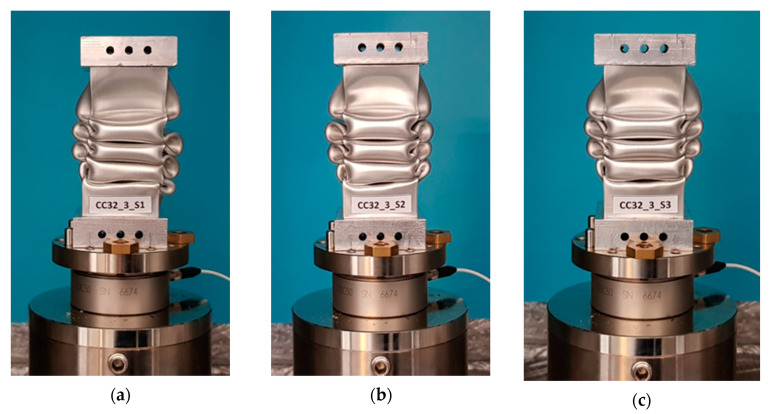
Specimens after the drop hammer test: (**a**) specimen CC32_3_S1, (**b**) specimen CC32_3_S2, (**c**) specimen CC32_3_S3.

**Figure 8:**
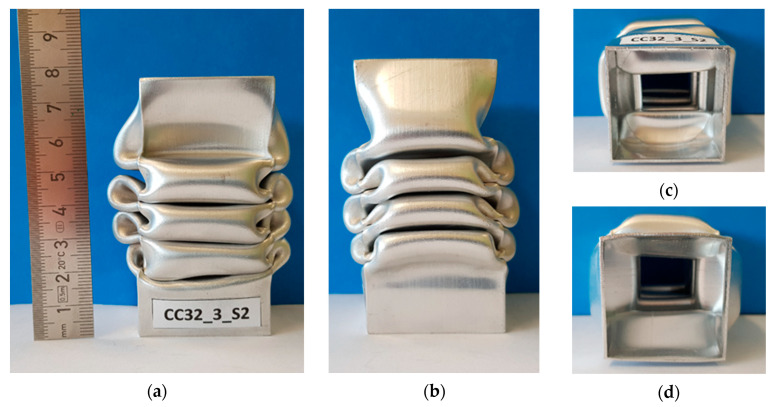
Exemplary post-failure deformation patterns of the tested specimen CC32_3_S2: (**a**) Front view, (**b**) side view, (**c**) bottom view and (**d**) top view.

The specimens in the machine after the test are shown in Figure 7, whereas Figure 8 presents a specimen viewed from different angles. Figure 7 shows the specimens after the test and Figure 8 shows specimen S2 in more detail.

Based on the obtained load-shortening characteristics the calculations for energy absorption indicators were conducted. These results were compared with the FEM calculations for a specimen with dimensions corresponding to the real specimens. The results of the calculations are listed in Table 2, where the percentage differences between the experimental tests and FEM calculations are also indicated. 

An analysis of the load-shortening characteristics showed great compliancy for all of the specimens in terms of the course and the values of shortening at which the entire energy was absorbed. The EA values were obtained as a field under a suitable characteristic and can insignificantly differ from the declared drop weight energy (both in the experiment and FEM). The S1 specimen became slightly askew during the test, which can be seen in Figure 7a. For this specimen, an increased peak of force during the occurrence of a fold over the trigger can be observed (Figure 6). The differences in the calculated values of the energy absorption indicators based on the data from the experiment and FEM calculations were insignificant, which was a result, among other things, of the appropriate adjustment of the mesh size.

Therefore, the study of the influence of geometric parameters of the indentations on the achieved indicators of energy absorption, which was carried out, showed the mark of reliability.

## 6. Finite Element Parametric Study—Results

**Figure 9:**
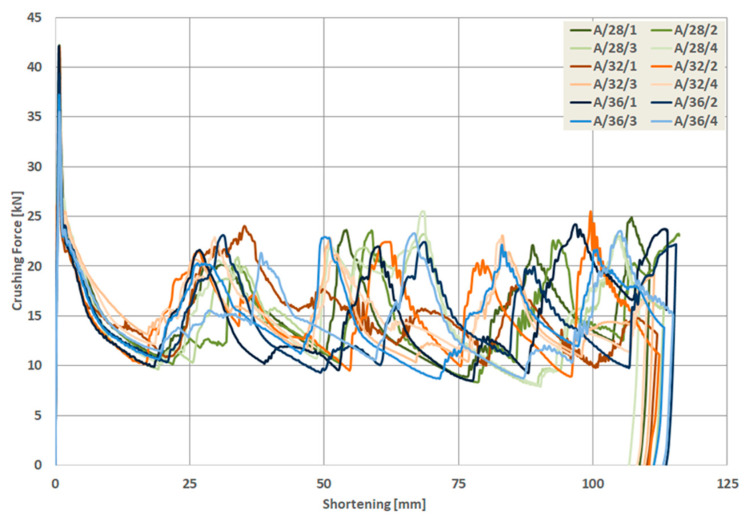
Load-shortening diagrams for columns A.

**Figure 10:**
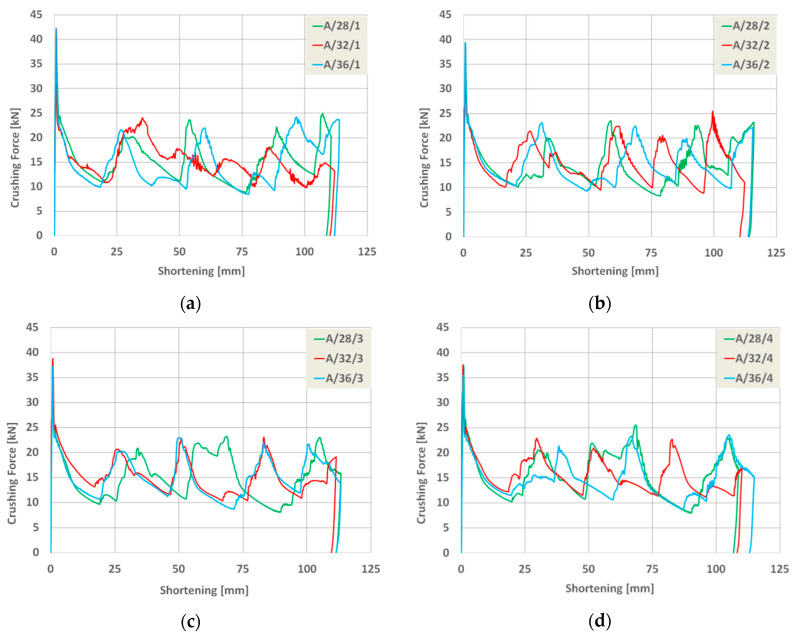
Load-shortening diagrams for columns A separately for variable depth of the indentation: (**a**) first group of models, (**b**) second group o models, (**c**) third group of models, (**d**) fourth group of models.

**Figure 11:**
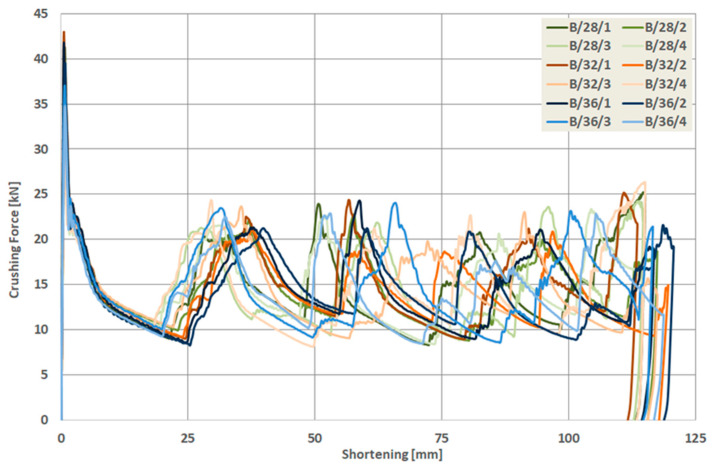
Load-shortening diagrams for columns B.

**Figure 12:**
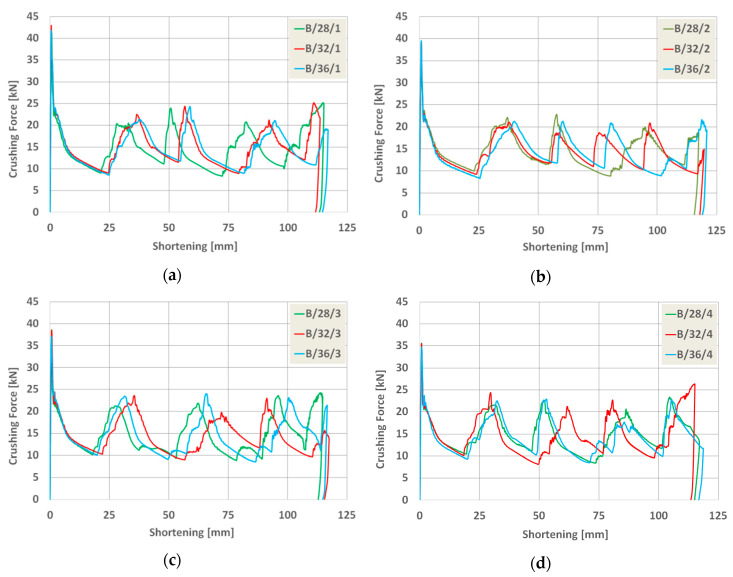
Load-shortening diagrams for columns B separately for variable depth of the indentation: (**a**) first group of models, (**b**) second group o models, (**c**) third group of models, (**d**) fourth group of models.

**Figure 13:**
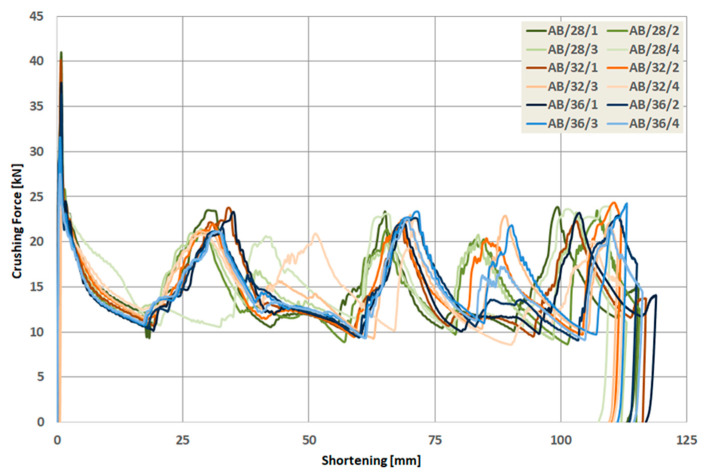
Load-shortening diagrams for columns AB.

**Figure 14:**
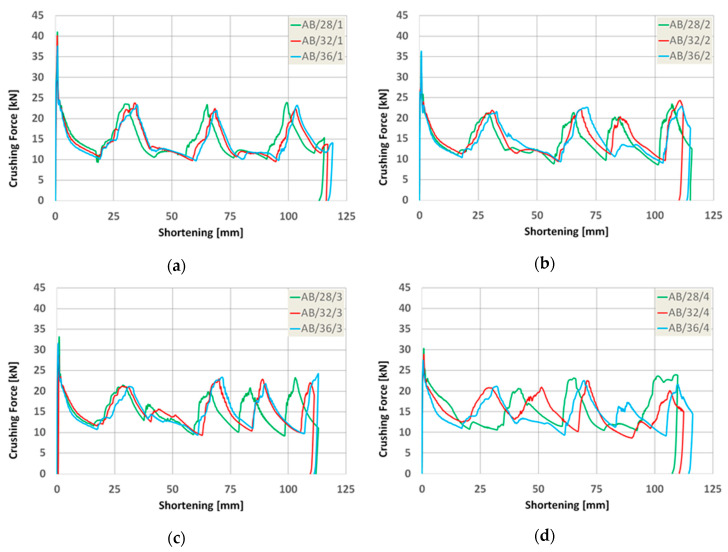
Load-shortening diagrams for columns AB separately for variable depth of the indentation: (**a**) first group of models, (**b**) second group o models, (**c**) third group of models, (**d**) fourth group of models.

As a result of the numerical analysis conducted in ABAQUS—Explicit software (Abaqus 2019, Dassault Systemes Simulia Corporation, Velizy Villacoublay, France), the characteristics of crushing force (shortening) for three groups of models were obtained. These characteristics are presented in Figure 9, Figure 10 and Figure 11, Figure 12 as well as Figure 13 and Figure 14. In order to render certain regularities of the model behavior during crash, the results of respective groups, presented separately for each indentation’s depth. The results were presented until the moment of energy ceased to 0. Once the impact energy ended, the specimens were subjected to elastic deformation and their height was insignificantly increased. 

Upon analyzing the course of the characteristics in Figure 9, Figure 10, Figure 11, Figure 12, Figure 13 and Figure 14, it was observed that all of those characteristics had a similar course. They had a typical course for crushing of tubular structures. At the beginning of the crushing process, an initial PCF appeared and then the crushing force oscillated around the average MCF force, which is also shown in Figure 2. These forces are shown in Table 3, Table 4 and Table 5 for each model. Regardless of the model group (A, B or AB) and the geometric parameters of the indentation, the course of the curves showed the same number of peaks, which indicated the same number of folds which formed during crushing. The differences in maximum specimen shortening (U) were 7.41 mm for A models, 7.15 mm for B models, and 8.98 for AB models.

However, after an in-depth analysis of the models divided by the depth of the indentation, one could draw a conclusion that along with the increase in the depth of the indentation, a more varied course occurred. This can be seen especially in the graphs concerning the AB model. At low indentation depth, the load-shortening process was almost identical regardless of the diameter (Figure 14a). As the depth of the trigger increased, differences in the course of the trigger began to appear, especially in the second half of the crush (Figure 14b,c).

When analyzing the characteristics of AB models with the deepest triggers, one could see significant differences in the course (Figure 14d). The case of AB/28/4 and AB/32/4 models required a separate analysis, since the course of load-shortening diagrams was different. Based on the conducted simulation, it was observed that the crash initiation occurred below the trigger. In the presented work, no damage initiation model was used in the simulation, but the elastic–plastic material model was used (properties of model are presented in Table 1).

**Figure 15:**
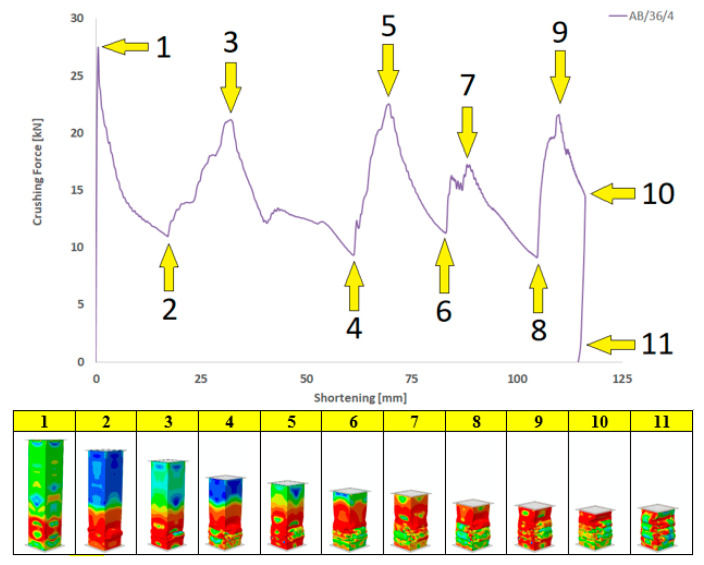
Characteristics of the crushing force (shortening) for the most beneficial model AB/36/4.

Figure 15 presents the characteristics of the crushing force (shortening) for the most beneficial model AB/36/4. Certain characteristic points on the chart were assigned their stage of structure deformation. The red color on the respective drawings, visualizing the crushing phases, show the zones where permissible stresses exceeded and plastic deformation occurred.

On the basis of the FE simulations, detailed parametric studies were carried out on the optimal depth and size of the indentation with regard to the energy absorption capacity for certain crash indicators referred to in Section 3. In the present work, four impact indicators were evaluated, namely, peak crushing force (PCF), mean crushing force (MCF), crash load efficiency (CLE), stroke efficiency (SE), and total efficiency (TE). The values of these indicators for the examined columns, based on the results of FE simulations, are shown in Table 3, Table 4 and Table 5. The results were subdivided according to the depth of the indentation in order to show certain regularities in a more distinctive way. The maximum shortening values (U) obtained for the moment when the speed of the tup hammer dropped to zero were also given.

Table 6 presents the abovementioned indicators obtained for a smooth column. These indicators will enable one to perform a comparison to assess the quantity of the advantages of using triggers in the form of spherical indentations.

**Figure 16:**
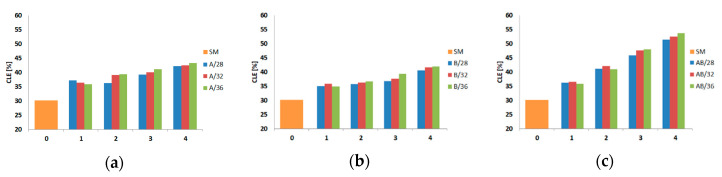
Crashworthiness indicator (crash load efficiency (CLE)) depending on the diameter and depth (abscissa) of the indentation: (**a**) A models, (**b**) B models, and (**c**) AB models.

**Figure 17:**
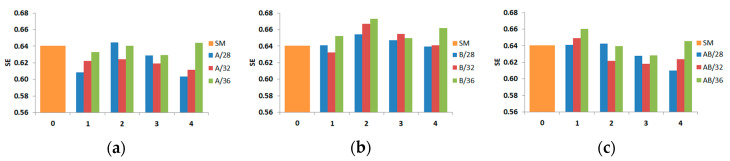
Crashworthiness indicator (stroke efficiency (SE)) depending on the diameter and depth (abscissa) of the indentation: (**a**) A models, (**b**) B models, and (**c**) AB models.

**Figure 18:**
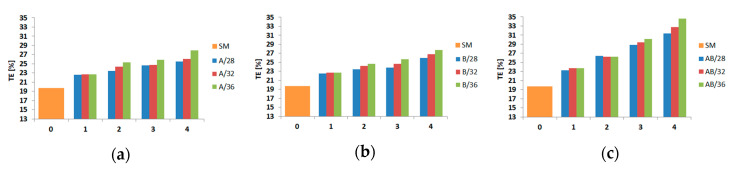
Crashworthiness indicator (total efficiency (TE)) depending on the diameter and depth (abscissa) of the indentation: (**a**) A models, (**b**) B models, and (**c**) AB models.

The calculated values of the energy absorption indicators are also presented in diagram form. Diagrams of three indicators (CLE, SE, and TE) are shown in Figure 16, Figure 17 and Figure 18.

Figure 16, Figure 17 and Figure 18 show only energy-absorbing indicators, but there is no information about EA, MCF, and PCF that could be of interest to scientists who are strictly interested in crashworthiness (EA, MCF, and PCF are presented in Table 3, Table 4, Table 5 and Table 6). The CLE indicator obtained ranged from 35.9% to 43.4% for A models and from 35.1% to 42.2% for B models depending on the diameter and depth of the trigger. The values were similar regardless of whether the indentation was concave or convex. The best energy-absorbing properties in the context of CLE values were achieved by AB models, where at an indentation deeper than 1, the increase in CLE compared with A and B models was already more pronounced. The maximum value of the indicator was 53.8% and was achieved by the model with the largest diameter and depth of the indentation. It is worth noting that all models had a CLE indicator higher than the smooth SM model, which achieved a value of about 30%.

Another SE indicator showed values between 0.604 and 0.645 for A models and between 0.61 and 0.66 for AB models. Only one or two models out of twelve examined in these groups showed SE indicator values higher than that calculated for the smooth column (SM). The situation was different in the group of B models, where 8 out of 12 models were characterized by a value higher than that obtained for the SM column. The SE values in this model group were strictly in the range from 0.63 to 0.66.

As far as the TE indicator was concerned, it could be seen that for all models, regardless of the diameter and depth of the trigger, an increase in value was obtained with reference to the value obtained for the SM column (19.46%). For models A and B, indicators ranging from 22.6% to 27.9% were obtained, while for models with a depth of 1, almost identical values were obtained regardless of the diameter. The maximum value of the indicator was 33.75%, which was 14% more than the value obtained for the SM (smooth) column.

## 7. Discussion

Passive safety issues have a practical impact on the solutions that will appear in the construction of crash boxes in cars, locomotives, etc. The novelty of this article is the shape of the trigger, which has not been considered so far and an attempt to answer the question which of the geometrical parameters of diameter or depth is more important in terms of increasing its energy absorption indicators compared to a smooth column. Calculations of energy-absorbing indicators allows to assess whether the new trigger solution is better or worse than the existing ones, provided that both energy absorbers have the same dimensions and are subjected to the same impact. The maximum CLE values for other triggers tested by us in the past are up to 45% [16] and they are much more difficult to implement in practice. The new trigger, which is the subject of this publication, reached a CLE of 54%, but its performance limits depend in practice on the formability of the aluminum wall and has not yet been fully determined. That means that if deeper embossing is possible, it will grow even more.

Upon conducting the study on the influence of the geometrical parameters of the indentation on the achieved energy absorption indicator, some general conclusions can be drawn. An analysis of three groups of models (A with convex indentations, B with concave indentations, and AB with convex and concave indentations on the opposite walls) showed that the best energy absorption properties were achieved by the models from the AB group. A good synergy of the applied convex and concave initiators occurred. The energy-absorbing properties of models A and B were similar. 

A very important conclusion was that the depth of the indentation had a significant influence on the indicators in all of the model groups. The diameter of the indentation had less significance. It was also observed that along with the increase in the depth of the indentation, the influence of the trigger diameter on the obtained CLE and, indirectly, TE indicators increased. In general, when it came to CLE and TE indicators, all models with triggers performed better than the smooth (SM) model. Since the best properties were achieved for the models with the deepest indentations, the limits of practical use are determined by the technological aspects connected to formability of aluminum. 

From this, it can be deduced that the depth of the indentation is a more important geometric parameter when it comes to energy-absorbing indicators. Models with shallow indentations achieve lesser results regardless of the size of the indentation. The worst results are produced by the shallow and extensive triggers. The model AB/36/4 was characterized by the best energy-absorbing parameters.

Energy absorbers differ not only in shape, dimensions, material, and thus some physical characteristics, but also in the energy they dissipate, exhausting their capacity within a certain strictly defined range. The comparative analysis between the models presented in the paper and other numerical models found in the subject’s literature was quite difficult from a quantitative point of view, but will be considered in the near future. This work focused primarily on the behavior during the crushing of a special type of columns and determination of energy-absorbing indicators for the models presented in this work. A measurable effect of future research, allowing for comparative analysis between the models presented by the authors and other similar solutions, will be the development of new energy-absorption indicators, which would allow for a clearer description of the behavior of thin-walled structures in terms of comparison with other models. In the future, it is planned to prepare a review paper concerning some kind of comparison (in some aspects) of the models presented in this and previous authors’ works with other similar solutions presented in the literature.

Further research related to the presented topics is also planned in the future, especially the influence of the distance from the base on the achieved indicators will be tested, as well as various materials and their strength and deformable properties will be considered.

## Figures and Tables

**Table 1:** Mechanical properties of the aluminum alloy AW6063-T6.

Young’s Modulus *E* (MPa)	70,000	Yield Strength (MPa)	200
Density, *ρ* (t/mm^3^)	2.7 × 10^−9^	Ultimate tensile strength (MPa)	272
Poisson’s ratio (-)	0.33	Elongation at break (%)	6

**Table 2:** Comparison of crashworthiness indicators for B/32/3 model.

Designations	EA (J)	U (mm)	PCF (kN)	MCF (kN)	CLE (%)	SE (-)	TE (%)
CC32 _3_S1	1710.7	114.2	39.70	14.99	37.8	0.634	24.0
CC32_3_S2	1707.5	115.5	37.30	14.78	39.6	0.642	25.4
CC32_3_S3	1706.5	115.3	39.72	14.79	37.2	0.641	23.8
Experiment (mean values)	1708.2	115.0	38.91	14.85	38.2	0.639	24.4
FEM	1711.9	117.5	38.43	14.57	37.9	0.653	24.7
Difference (%)*	0.2	2.2	1.2	1.9	0.8	2.2	1.4

* Relative difference with respect to experimental values.

**Table 3:** Crashworthiness indicators and maximum shortening for A models.

Model	EA (J)	U (mm)	PCF (kN)	MCF (kN)	CLE (%)	SE (-)	TE (%)
A/28/1	1715.5	109.56	42.13	15.66	37.17	0.609	22.62
A/32/1	1714.1	111.96	42.00	15.31	36.45	0.622	22.68
A/36/1	1713.9	113.89	41.88	15.05	35.93	0.633	22.74
A/28/2	1717.4	116.04	40.74	14.80	36.33	0.645	23.42
A/32/2	1715.9	112.34	39.08	15.27	39.09	0.624	24.40
A/36/2	1720.6	115.28	37.83	14.93	39.46	0.641	25.27
A/28/3	1717.3	113.18	38.65	15.17	39.26	0.629	24.69
A/32/3	1715.4	111.41	38.44	15.40	40.06	0.619	24.79
A/36/3	1717.2	113.25	36.82	15.16	41.18	0.629	25.91
A/28/4	1716.4	108.63	37.46	15.80	42.18	0.604	25.46
A/32/4	1712.4	110.12	36.54	15.55	42.55	0.612	26.03
A/36/4	1715.5	115.93	34.13	14.80	43.36	0.644	27.93

**Table 4:** Crashworthiness indicators and maximum shortening for B models.

Model	EA [J]	U [mm]	PCF [kN]	MCF [kN]	CLE [%]	SE [-]	TE [%]
B/28/1	1715.6	115.12	42.21	14.90	35.31	0.640	22.58
B/32/1	1714.1	113.57	41.90	15.09	36.02	0.631	22.73
B/36/1	1716.0	117.09	41.79	14.66	35.07	0.651	22.81
B/28/2	1716.2	117.48	40.57	14.61	36.01	0.653	23.50
B/32/2	1715.9	119.64	39.33	14.34	36.46	0.665	24.24
B/36/2	1715.8	120.72	38.51	14.21	36.90	0.671	24.75
B/28/3	1717.1	116.15	39.98	14.78	36.98	0.645	23.86
B/32/3	1711.9	117.53	38.43	14.57	37.90	0.653	24.75
B/36/3	1715.9	116.63	37.04	14.71	39.72	0.648	25.73
B/28/4	1716.9	114.81	36.59	14.96	40.87	0.638	26.07
B/32/4	1712.6	115.11	35.44	14.88	41.98	0.640	26.85
B/36/4	1714.5	118.75	34.20	14.44	42.22	0.660	27.85

**Table 5:** Crashworthiness indicators and maximum shortening for AB models (models subdivided by the depth of the indentation).

Model	EA (J)	U (mm)	PCF (kN)	MCF (kN)	CLE (%)	SE (-)	TE (%)
AB/28/1	1717.0	115.43	40.90	14.87	36.37	0.641	23.32
AB/32/1	1716.1	116.90	40.15	14.68	36.57	0.64	23.75
AB/36/1	1716.8	118.91	40.17	14.44	35.94	0.661	23.75
AB/28/2	1718.0	115.65	36.04	14.86	41.22	0.643	26.48
AB/32/2	1715.8	111.97	36.28	15.32	42.23	0.622	26.27
AB/36/2	1718.1	115.10	36.33	14.93	41.09	0.639	26.27
AB/28/3	1717.3	113.05	33.04	15.19	45.98	0.628	28.88
AB/32/3	1708.9	111.27	32.19	15.36	47.70	0.618	29.49
AB/36/3	1718.4	113.11	31.54	15.19	48.17	0.628	30.27
AB/28/4	1716.4	109.83	30.35	15.63	51.48	0.610	31.42
AB/32/4	1707.4	112.33	28.90	15.20	52.60	0.624	32.82
AB/36/4	1716.8	116.20	27.45	14.77	53.82	0.646	34.75

**Table 6:** Crashworthiness indicators and maximum shortening for the SM model.

Model	EA (J)	U (mm)	PCF (kN)	MCF (kN)	CLE (%)	SE (-)	TE (%)
SM	1717.1	115.86	49.04	14.83	30.23	0.644	19.46
